# Human Dental Pulp Stem Cells Hook into Biocoral Scaffold Forming an Engineered Biocomplex

**DOI:** 10.1371/journal.pone.0018721

**Published:** 2011-04-11

**Authors:** Carlo Mangano, Francesca Paino, Riccardo d'Aquino, Alfredo De Rosa, Giovanna Iezzi, Adriano Piattelli, Luigi Laino, Thimios Mitsiadis, Vincenzo Desiderio, Francesco Mangano, Gianpaolo Papaccio, Virginia Tirino

**Affiliations:** 1 Department of Biomaterials Science, Università dell'Insubria, Varese, Italy; 2 Section of Histology and Embryology, Tissue Engineering and Regenerative Medicine (TERM) Division, Department of Experimental Medicine, Second University of Naples, Naples, Italy; 3 Department of Odontology, Second University of Naples, Naples, Italy; 4 Department of Odontology, University “G. D'Annunzio” Chieti-Pescara, Chieti, Italy; 5 Institute of Oral Biology, University of Zurich, Zurich, Switzerland; Brigham & Women's Hospital - Harvard Medical School, United States of America

## Abstract

The aim of this study was to evaluate the behavior of human Dental Pulp Stem Cells (DPSCs), as well as human osteoblasts, when challenged on a Biocoral scaffold, which is a porous natural hydroxyapatite. For this purpose, human DPSCs were seeded onto a three-dimensional (3D) Biocoral scaffold or on flask surface (control). Either normal or rotative (3D) cultures were performed. Scanning electron microscopic analyses, at 8, 24 and 48 h of culture showed that cells did not adhere on the external surface, but moved into the cavities inside the Biocoral structure. After 7, 15 and 30 days of culture, morphological and molecular analyses suggested that the Biocoral scaffold leads DPSCs to hook into the cavities where these cells quickly start to secrete the extra cellular matrix (ECM) and differentiate into osteoblasts. Control human osteoblasts also moved into the internal cavities where they secreted the ECM. Histological sections revealed a diffuse bone formation inside the Biocoral samples seeded with DPSCs or human osteoblasts, where the original scaffold and the new secreted biomaterial were completely integrated and cells were found within the remaining cavities. In addition, RT-PCR analyses showed a significant increase of osteoblast-related gene expression and, above all, of those genes highly expressed in mineralized tissues, including osteocalcin, OPN and BSP. Furthermore, the effects on the interaction between osteogenesis and angiogenesis were observed and substantiated by ELISA assays. Taken together, our results provide clear evidence that DPSCs differentiated into osteoblasts, forming a biocomplex made of Biocoral, ECM and differentiated cells.

## Introduction

Bone grafting to replace missing bone with synthetic porous Biomaterial (i.e. bone graft scaffolds) and associated new bone formation and remodelling, have been investigated for over 30 years [1]. Limited availability of autografts and the risk of disease transfer of allografts, however, has produced an increase in requests for synthetic bone grafts.

Novel approaches for bone substitutes are focused on stimulation of osteointegration, osteoconduction, osteoinduction as well as induction of angiogenesis and vascularisation, by designing bioactive materials with appropriate pore architecture [1].

On the other hand, the scaffolds used in tissue engineering for bone regeneration must also act as a template for cell adhesion, migration, proliferation, cell to cell interactions and the formation of bone-extracellular matrix, providing structural support to the newly formed tissue.

In addition, they can serve as delivery vehicles for cytokines such as bone morphogenetic proteins (BMPs), insulin-like growth factors (IGFs) and transforming growth factors (TGFs) that stimulate recruited host precursor cells to differentiate into bone-matrix producing cells [2], thus providing osteoinduction. Finally, scaffolds for osteogenesis should have an interconnected porosity so as to help tissue integration and vascularisation.

Porous scaffolds are thought to have all these features: they are utilized to induce good bone healing by three-dimensional tissue growth.

Due to their interconnected porous architecture, high compressive breaking stress, good biocompatibility and reabsorbability, corals have been used as scaffolds for bone tissue engineering. Transcortical bony defects implanted with coral become vascularised and are invaded by newly formed bone, whereas the coral is reabsorbed at a rate commensurate with bone formation [3].

It has been postulated that greater regeneration could be obtained by supplementing a reabsorbable scaffold with osteogenic cells such as bone marrow stromal cells (BMSCs) or umbilical cord-derived stem cells to improve clinical outcome [4], [5], [6]. Stem cell–based tissue engineering has been shown to be highly advantageous in bone regeneration when adult mesenchymal stem cells (MSCs) are used.

Therefore taking into consideration that human dental pulp stem cells (DPSCs) are MSCs that quickly differentiate into osteoblasts and endothelial cells both *in vitro* and *in vivo* [7], [8], and are able to successfully repair human bone defects [9] we decided to use these cells, and primary human osteoblasts for comparison, to observe their capabilities on a Biocoral scaffold.

Our main objective was to study the osteogenic differentiation of DPSCs or human osteoblasts, challenged on a Biocoral scaffold, as well as to provide fundamental information allowing the use of DPSCs/Biocoral as a suitable engineered biocomplex, made of human mesenchymal-derived cells and a porous structure, for defect repair or transplantation purposes.

## Results

### Cell growth analysis

Cell growth analysis and viability staining with trypan blue showed that the scaffold did not show significant effects on the viability of each cell population with respect to its control (flask surface).

The cell doubling time was of 45±0.29 hours for DPSCs seeded in flasks and of 45.7±0.18 hours for DPSCs grown on scaffolds. For osteoblasts, the doubling time was of 34±0.15 hours for cells seeded in flasks versus 33.8±0.25 for those grown on Biocoral.

### Cultures and scanning electron microscopy

**Figure 1:**
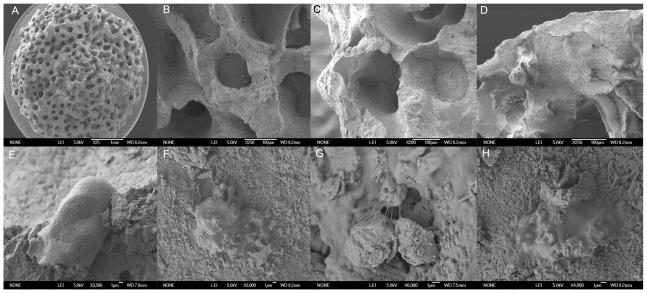
Scanning electron microscopy (SEM) micrographs. This figure shows the Biocoral scaffold structure at different magnification (see scale bars at the bottom of each micrograph) (A–D) evidencing size and regularity of porosity and pore interconnections. SEM micrographs showing (E–F) DPSCs and (G–H) human osteoblasts grown on Biocoral scaffold at 8 h and 24 h after seeding. Cell adhesion and spreading on scaffold porosity can be observed.

DPSCs isolated from the dental pulps of healthy individuals were sorted for c-kit/CD34/flk-1. Both primary osteoblasts and DPSCs were seeded on Biocoral scaffolds, as well as on flasks (flask surfaces) in standard cultures and then processed for scanning electron microscope (SEM) analysis at 4, 8 and 24 h. Cell density and morphology on both surfaces were investigated. On Biocoral surfaces, a very small number of cells (either DPSCs or osteoblasts) was detectable at the times considered (data not shown). This can be due to the fact that cells that quickly moved into the cavities inside the Biocoral, rich in depressions and deep, rounded pits of widely varying shape and size (Fig. 1A–D). In Biocoral cavities, cells were attached to the surface and did not show a flattened or spherical shape (Fig. 1 E–H). Cells seemed to reach a stabilized shape only after 8 h of culture, although DPSCs (Fig. 1 E,F) and primary osteoblasts displayed osteoblastic features at 24 h (Fig. 1 G, H).

### Histological evaluation

**Figure 2:**
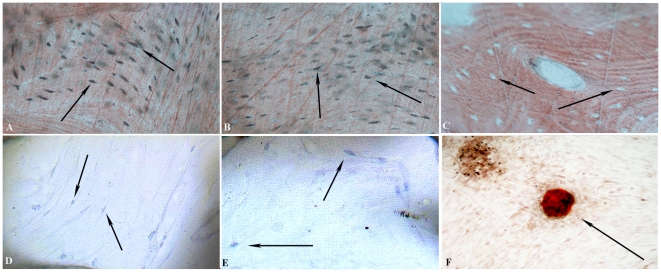
Histological sections. Light microscopic images showing the Biocoral scaffold that is (A) loaded with DPSCs after 30 days of 3D culture and stained with Alizarin red and Toluidine Blue or (D) only with Toluidine Blue, showing the red staining of bone and the blue staining of cells (*arrows*) that are completely entrapped within the Biocoral cavities; or (B) loaded with human osteoblasts after 30 days of 3D culture and stained with Alizarin red and Toluidine Blue or (E) only with Toluidine Blue, also showing the red staining of bone and the blue staining of entrapped cells (*arrows*), inside the Biocoral cavities; (C) Alizarin red and Toluidine blue staining of Biocoral without cells. Arrows indicate Biocoral cavities without cells; (F) the Alizarin red stains a calcification nodule (*arrow*) obtained from DPSCs cultured for 30 days on a flask surface (control). Original magnification 400×.

Histological sections of DPSCs and osteoblasts seeded on Biocoral showed a diffuse bone formation within this scaffold, although it was impossible to establish the exact amount of new bone deposition, because it was disseminated within the internal cavities (Fig. 2 A,B). In the control sample (Fig. 2C), being the Biocoral made of hydroxyapatite and having a bone-like structure, it displays the same Alizarin red staining of mineralized structures detectable for both DPSCs and osteoblasts. The cells were clearly observable in all the samples, inside the structure and up to the surface of the pores (Fig. 2 A,B). In particular, it was possible to observe that cells were located inside the cavities.

Toluidine blue staining confirmed the presence of cells and their colonization inside the Biocoral, entrapped in it (Fig. 2 C). On flask surfaces, mineralized bone nodules formation was observed and stained with Alizarin red (Fig. 2D).

### Expression of bone-associated genes

To evaluate the effects of the Biocoral scaffold on osteogenic differentiation, we performed RT-PCR analyses for bone-associated genes in DPSC cultures either grown on Biocoral scaffold, or on a flask surface, at different times of their differentiation (7-15-30 days). Osteoblasts grown in the same conditions, as mentioned above, were used as controls.

**Figure 3:**
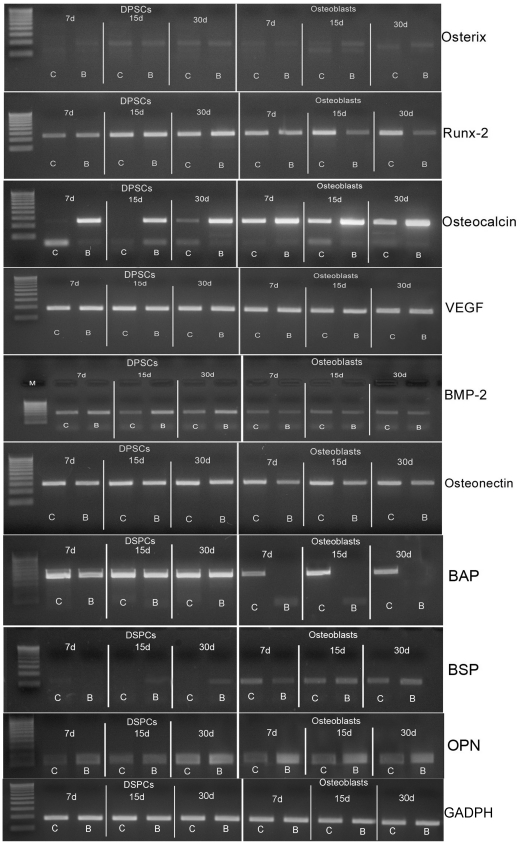
RT-PCR analyses for osteoblast-specific genes. The analyses were performed on the following genes: Osterix, Runx-2, Osteocalcin, VEGF, BMP-2, osteonectin, BAP, BSP, osteopontin (OPN). It is possible to observe that at 7, 15 and 30 days of culture cells grown on flask surface (lane C) or on Biocoral (lane B) were analyzed for the expression of early, middle and late stage osteoblast differentiation genes. See Table 1 and 2 for quantification data.

RT-PCR analyses (Fig. 3 and Tables 1 and 2) revealed that no differences for Runx-2 expression were detectable in DPSCs seeded both on the Biocoral scaffold and on flask surfaces, although its expression increased from day 7 to day 15. Conversely, in osteoblasts, Runx-2 expression considerably decreased in cells seeded on Biocoral scaffold.

**Table 1:** Densitometry measurement of mRNA level for DPSCs sedeed on Biocoral scaffold (B) and flask surface(C).

Day	Osterix		Runx-2		Osteocalcin	
	C	B	C	B	C	B
**7**	0.449±0.11	5.776±0.92**	4.380±0.91	6.114±1.12	0.0426±0.03	1.070±0.24**
**15**	12.2±0.53	12.456±0.45	10.325±0.70	11.125±0.68	0.055±0.04	0.953±0.13**
**30**	12.548±0.64	12.460±0.59	10.014±0.82	12.286±0.95	0.181±0.09	1.019±0.15**

	VEGF		BMP-2		Osteonectin	
	C	B	C	B	C	B
**7**	7.891±1.15	9.543±0.92	10.638±0.24	14.509±0.35*	9.197±0.79	8.069±0.93
**15**	9.697±0.81	8.540±0.63	5.104±0.16	15.865±0.98**	10.514±0.81	9.364±1.54
**30**	8.665±0.27	9.059±0.12	8.406±0.31	15.490±0.43**	11.070±1.06	9.558±1.87

	BAP		BSP		Osteopontin	
	C	B	C	B	C	B
**7**	13.860±0.12	10.025±2.89	1.661±0.01	1.665±0.01	2.508±0.37	6.748±0.45**
**15**	12.697±0.79	11.572±1.81	3.641±0.58	5.901±0.34*	4.915±0.79	6.035±0.13
**30**	11.589±0.15	11.810±0.12	5.090±0.25	8.646±0.18*	11.997±0.26	14.351±0.31*

*p<0.005.

**p<0.001.

**Table 2:** Densitometry measurement of mRNA level for osteoblasts sedeed on Biocoral scaffold (B) and flask surface(C).

Day	Osterix		Runx-2		Osteocalcin	
	C	B	C	B	C	B
**7**	9.452±1.01	6.858±0.95	9.554±1.4	8.133±2.01	0.890±0.12	1.090±0.23*
**15**	7.284±0.62	7.510±1.23	11.766±0.81	3.656±0.54**	0.780±0.14	1.134±0.35*
**30**	6.094±0.76	6.905±0.45	10.136±0.34	2.510±0.92**	0.754±0.28	1.104±0.35*

	VEGF		BMP-2		Osteonectin	
	C	B	C	B	C	B
**7**	8.421±0.67	8.477±0.86	5.388±0.53	3.346±1.98	8.392±0.43	4.799±0.56*
**15**	7.400±0.89	7.996±1.21	6.059±0.48	4.580±1.03	9.332±0.67	6.494±0.55*
**30**	7.271±0.97	7.041±1.11	5.942±0.93	4.672±1.24	7.910±0.71	5.301±0.49*

	BAP		BSP		Osteopontin	
	C	B	C	B	C	B
**7**	5.352±0.67	0.641±0.13**	13.961±1.10	6.790±3.76	4.875±0.87	14.471±1.14**
**15**	11.216±0.84	3.104±0.12**	12.198±0.92	14.796±0.75*	6.939±0.79	11.622±1.11**
**30**	7.820±1.3	0.316±0.21**	11.187±0.74	14.464±1.23*	5.651±0.64	9.888±0.89**

*p<0.005.

**p<0.001.

Osterix was another marker of osteoblast differentiation that we considered in our observations. Actually, its expression showed a slight but progressive increase from day 7 to day 15 in both DPSCs and osteoblasts.

Bone Alkaline Phosphatase (BAP) was highly expressed by DPSCs on both Biocoral and flask surfaces, while osteoblasts showed a lack of expression, when cultured on Biocoral scaffold.

The expression of Osteopontin, which is considered one of the major sialoprotein of the extracellular matrix of bone and serves as a bridge between cells and hydroxyapatite [10], was increased at day 30 in DPSCs and already at day 7 in osteoblasts grown on Biocoral scaffold.

Osteonectin, a glycoprotein that binds calcium and that is secreted by osteoblasts during bone formation, initiating mineralization and promoting mineral crystal [11], was shown to be considerably expressed in all DPSCs samples at the same levels, while ii was slightly decreased in osteoblasts grown on Biocoral scaffold.

Bone sialoprotein (BSP), a significant component of the bone extracellular matrix was slightly expressed by DPSCs, but its expression was up-regulated starting from day 15 on Biocoral scaffold. The same results were detected in osteoblasts.

Noteworthy, the expression of osteocalcin, which is a late stage marker of osteogenic differentiation, was found to be strongly increased in the presence of the Biocoral scaffold in both DPSCs and osteoblasts, with respect to their controls during the osteoblast differentiation.

Moreover, we analyzed also BMP-2 and VEGF expression. Regarding BMP-2 expression, it was significantly up-regulated in DPSCs seeded in the Biocoral scaffold, with respect to DPSCs seeded in the flask surfaces. In osteoblasts no significant differences were detectable.

RT-PCR analyses revealed that VEGF, which is a potent angiogenic stimulator that plays an important role during bone formation, was highly expressed in all the samples.

### ELISA assays

**Figure 4:**
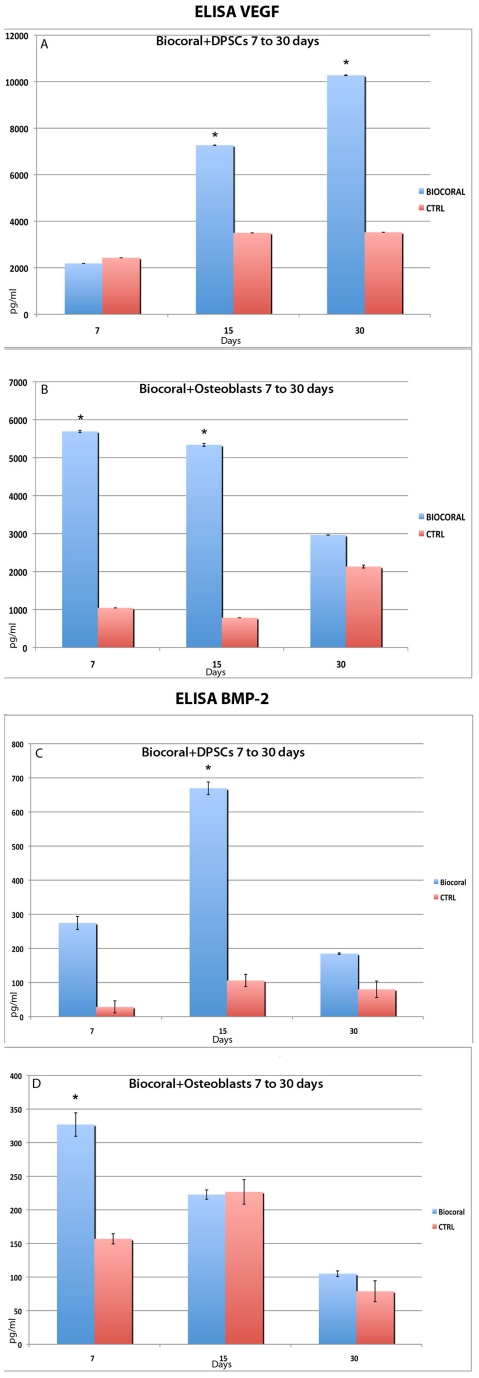
ELISA assays for BMP-2 and VEGF protein secretion. These observations were performed on DPSCs and human osteoblast secretions at 7, 15 and 30 days of culture. Data are given as average values ±SD. *p<0.001.

ELISA assays (Fig. 4) were performed on culture media in order to detect the amounts of VEGF and BMP-2 growth factors at 7, 15 and 30 days. The time steps were chosen in order to understand the kinetics of the relative amounts of both morphogens secreted by the cells (DPSCs or osteoblasts) in both conditions: seeded and cultured either on the Biocoral scaffold or on a flask surfaces.

#### VEGF

The amount of VEGF secreted by DSPCs considerably and significantly increased from 7 up to 30 days on Biocoral scaffold (p<0.01) (Fig. 4A); interestingly, the amounts of VEGF secreted by cells seeded on Biocoral were significantly higher than those found in samples on flask surfaces (p<0.01) (Fig. 4A) .

On the other hand, VEGF amounts secreted by osteoblasts on flask surfaces were found to be significantly decreased from day 15 up to day 30. In addition, the VEGF amount belonging to Biocoral samples, when compared with the amounts belonging to osteoblasts in flask surface, were found to be significantly higher (p<0.01). (Fig. 4B)

#### BMP-2

From 7 to 30 days, while BMP-2 amounts released by DSPCs on flask surfaces were found to be of low amounts, those secreted by cells cultured with Biocoral scaffolds were found to be significantly increased up to 15 days (p<0.01), although they had then dropped at day 30 (Fig. 4 C).

In addition, the amounts of BMP-2 levels secreted by osteoblasts on Biocoral were much higher (p<0.01) than those secreted by osteoblasts on flasks at day 7, then these levels tended to decrease by day 15 days of culture, up to day 30 (Fig. 4 D).

## Discussion

Up to a few years ago, the gold standard for bone grafting surgical procedures was considered to be the use of autologous bone grafts, capable to stimulate bone growth and implant fixation. However, limited amounts of bone are available for autografting and the harvesting procedures involve high donor site morbidity [1]. Allografts from bone banks are readily available, but the complication rate is high with the risks of graft-versus-host disease, transmission of infectious diseases and graft failure [12], [13], [14]. Therefore, alternative methods including those using stem/progenitor cells have been recently investigated [7], [8], [15] and human trials have already been successfully carried out [9].

Actually, in bone tissue engineering, mainly regarding large defects, a possible strategy involves the culture of osteogenic cells on porous scaffolds. Osteogenesis occurs by seeding the scaffolds before implantation with cells that will establish new centres for bone formation [2], such as osteoblasts and mesenchymal cells that have the potential to commit to an osteoblastic lineage. The aim is to achieve a tool for bone grafting by using autologous mesenchymal stem cells (MSC). Human MSCs (hMSCs) are an attractive cell source because they are readily extracted from several sites in humans such as adipose tissue, dental pulp or bone marrow [4], [5], [6]. Among the hMSCs, dental pulp stem cells have been demonstrated to be ideal for their use in bone differentiation and tissue engineering [7], [8], [9], [15] .

Natural coral exoskeleton has the best mechanical properties of the porous calcium-based ceramics, given that its pores range from 150 to 600 µm in diameter, with interconnecting pore sizes averaging approximately 260 µm in diameter. These dimensions are similar to that of spongy bone, thus making coral an excellent base for the spread of new bone ingrowth. In addition, calcium-phosphate materials can be technologically modified, chemically and structurally designed with specific architecture, forms, and geometry [16] . All of these properties could drive cell functions, forcing cells to express the desired osteogenic phenotype [17] .

On the other hand, we have recently demonstrated [18] that a sintered titanium surface, due to its specific topographical characteristics, allows DPSCs to quickly differentiate into osteoblasts and produce considerable amounts of bone with respect to other titanium surfaces (machined) meaning that the scaffold's surface is of paramount importance for cell differentiation and fate.

In the present study we show that stem cells quickly move within Biocoral cavities and then they start to differentiate into osteoblasts. Differentiation and matrix mineralization *in vitro* involve a considerable expression of genes as well as protein production, leading to the mineralization.

Our findings demonstrate that Biocoral scaffold induced an increase osteoblast-related gene expression in DPSCs. In fact, we observed an increase in mRNA expression of the bone-associated transcription factors, like Osterix and Runx-2 and a strong up-regulation of osteocalcin, a marker of late-stage osteoblast differentiation. These findings suggest that DPSCs are differentiating into osteoblasts.

Furthermore, to assess the effects on mineral formation, we decided to detect BSP and OPN mRNA expression and we found that both BSP and OPN mRNAs were up-regulated in DPSCs culture grown on Biocoral. Taking into consideration that formation of mineralized matrix is a definitive hallmark of osteoblastic differentiation, we can assume that Biocoral scaffold stimulates osteoblast differentiation and function inducing matrix mineralization as confirmed also by Alizarin Red staining.

On the other hand, the osteoblast cell culture, that we used as a control, underwent bone differentiation, since these cells are already self-committed. In fact, osteoblasts cultured on the Biocoral scaffold showed a high expression of the late-stage differentiation markers, including OPN, BSP and osteocalcin, whereas the expression of early-stage markers, like Runx-2, BMP-2 and BAP, decreased. The findings can be also explained considering that, as it is well known, the expression of Runx-2 and BAP in bone formation has an important role in the early and middle stages of differentiation, while the expression of BSP, OPN and osteocalcin has a significant role in the late stages of osteoblast differentiation.

In addition, in this study, we have assayed the amount of morphogens secreted by stem cells and osteoblasts by ELISA assay. Our results show that the amount of BMP-2 and VEGF secreted by DPSCs seeded on Biocoral scaffold were increased with time, with respect to the already differentiated cells, thereby establishing the interaction between osteoblast function and angiogenesis which are combined, closely associated processes leading to bone formation [7], although these data do not agree with RT-PCR analyses. Post-transcriptional regulations of gene expression enable cells to control the contents of their proteomes. Therefore, the protein production does not always show the same trend of m-RNA expression. Post-transcriptional regulations could be occurred justifying different results between RT-PCR and ELISA for VEGF analyses. The latter may explain the differences that we have found and described.

In conclusion, with this study we provide clear evidence that stem cells (namely DPSCs) differentiating into osteoblasts lead to a biomineralization, supported by the bioscaffold surface, that is capable to induce a rather complex gene expression as well as bone morphogenetic protein production. The biocomplex made of osteoblasts originating from dental pulp stem cells and Biocoral seems to be a reliable new tool for bone tissue engineering.

## Materials and Methods

### Dental pulp extraction and digestion

Human dental pulp was extracted from third molar teeth of 8 healthy adults with written informed consent, approved by our Internal Ethical Committee (Second University Ethical Committee) and following our protocol [8]. Before extraction, each subject was checked for systemic and oral infection or diseases. Only disease-free subjects were selected. Each subject was pretreated for a week with professional dental hygiene. Before extraction, the dental crown was covered with 0.3% chlorhexidine gel (Forhans, NY, USA) for 2 min and then the pulp was extracted with a dentinal excavator or a Gracey curette.

### Dental pulp stem cell and primary osteoblasts cultures

Once removed, each pulp was immersed in a digestive solution (type I collagenase 3 mg/ml plus dispase 4 mg/ml in phosphate buffer saline, PBS, containing 100 U/ml penicillin, 100 mg/ml streptomycin) for 1 h at 37°C in agitation. The solution was then filtered through 70 µm Falcon strainers (Becton and Dickinson, Franklin Lakes, NJ, USA). After filtration, cells were pooled and immersed in MegaCell Minimum Essential Medium (Sigma, Milan, Italy) supplemented with 10% FBS, 100 mM 2P-ascorbic acid, 2 mM L-glutamine, 100 U/ml penicillin, 100 mg/ml streptomycin (all purchased from Invitrogen, San Giuliano Milanese, Milan, Italy) and placed in 75 ml flasks with filtered valves. Flasks were incubated at 37°C and 5% CO_2_ and the medium changed twice a week. Just before cells become confluent, they were subdivided into new flasks. Stem cells were sorted (see below) only when their number was at least 1,000,000 cells per flask. This number was achieved around day 22, when they were still undifferentiated. Differentiated cells were obtained from sorted stem cells cultured for at least 30 days in α-MEM culture medium with 20% FBS (all purchased from Invitrogen, San Giuliano Milanese, Milan, Italy); in fact, FBS promotes osteoblastic differentiation when used at a high percentage, as we have previously demonstrated [8], [19].

Human osteoblasts were obtained from cortical mandible of healthy patients free of bone-related disease, who underwent of third molar extraction. Bone chips were rinsed three times in phosphate buffered saline (PBS), broken into small pieces and cultured in alpha-MEM supplemented with 10% FBS. Primary osteoblast cultures were kept in a humidified atmosphere of 5% CO_2_ at 37°C. Both DPSCs and primary osteoblasts were seeded onto Biocoral scaffolds of constant size (5 mm diameter and 1,5 mm thick); 200 µl of 10^5^cells/ml were seeded onto each scaffold in 24-well plates with a sterile syringe. Cells were incubated for 2 hours at 37°C in 5% CO_2_ atmosphere before adding the culture medium to a total volume of 1 ml.

### Biocoral Scaffold

The calcium-carbonate coral-derived material (Genus Porites) evaluated in this study presents a chemical composition very similar to human bone [20]. Biocoral consists of more than 98% calcium carbonate in crystal form (aragonite) and other elements (F and Sr 0.7–1%, Mg 0.05–0.2%,Na<1%, K<0.03%, P<0.05%, water<0.5%, and amino acids<0.026%). Among all these elements, the presence of strontium is fundamental, as it can effectively promote mineralization processes [21]. Biocoral is biocompatible and osteoconductive and interestingly, it possesses an average porosity of 50% and it is similar to cancellous bone, with an architecture composed by strongly interconnected pores of variable diameter (250–500 µm).

### Tissue engineering and rotating cultures

In order to achieve 3-dimensional tissue formation, we challenged DPSCs and primary osteoblasts in a roller apparatus (Wheaton, Millville, NJ) with Biocoral scaffold.

At least 500,000 cells after sorting were gently plated onto 3-dimensional scaffolds made of Biocoral. Samples were placed in the roller apparatus and left for 30 days at a speed of 6 rpm in an incubator at 37°C and 5% CO_2_. At the end of the experiments, all the specimens were processed for histological observations, as specified below.

### FACScanning, sorting and differentiation

Cells were sorted using both morphological traits (high side scatter and low forward scatter) and antigenic criteria (firstly using CD117 and CD34, and then flk-1), as specified previously [7], [8], [9], [19]. Only cells that co-expressed all these markers (5% of total cell population) were sorted in order to obtain a homogeneous population, called DPSC. Briefly, cells were detached using 0.02% EDTA in PBS and pelleted by centrifugation (10 min at 1,000 rpm), washed in PBS at 0.1% BSA at 4°C and incubated with 1 µg/ml of antibody. Cells were washed in the same solution once and were processed for sorting (FACsorter ARIA II, Becton & Dickinson, Franklin Lakes, NJ, USA). The mouse anti-human antibodies CD117 (c-kit), CD34, and flk-1 were purchased from DBA, Segrate, Milan, Italy.

For indirect immunofluorescence cytometric assay, FITC and PE-labeled goat anti-mouse (Santa Cruz) were used. The purity of sorted populations was routinely 90%. Isotype antibodies were used as controls.

Osteogenic differentiation was achieved as previously reported [7], [8], [9]. Briefly, SBP-DPSCs were cultured with 20% FBS for 15 days without passaging, after which cells were cultured with 20% FBS. We have extensively demonstrated that SBP-DPSCs do not require dexametasone or addition of other substances to achieve osteodifferentiation and bone production (Laino et al, 2005; 2006, d'Aquino et al., 2007). To monitor differentiation, the cells were examined using mouse anti-human antibodies to CD44, the transcription factor Runx-2 (all from Santa Cruz, CA, USA). For Runx-2 analysis, cells were processed using the Caltag Fix & Perm Kit (Invitrogen, Milan, Italy) following the manufacturer's guidelines. Isotypes were used as controls. All data were analyzed using CellQuest software.

### Cell growth analysis

In order to assess the scaffold's ability to sustain cell growth and expansion (of both DPSCs and osteoblasts), growth curves for cells seeded on scaffolds versus cells seeded on flask surfaces were compared. For this purpose, cells were seeded in equal densities in 24 well-plates (see above) and harvested by trypsinization. After washing in PBS, the cell suspension was diluted 1∶1 with 0.4% trypan blue solution (Sigma–Aldrich), and viable and non-viable cells were counted using the hemocytometer chamber under an inverted microscope. Cells from triplicate samples were considered at 24 h intervals for 10 days. The average cell count of each day was used to determine growth curves and doubling time.

### Scanning electron microscope (SEM)

Cells were fixed in 2.5% glutaraldehyde (EM grade) in 0.1 M phosphate buffer, postfixed in 0.1% OsO_4_ in the same buffered solution for 1 h and, after critical point drying and gold-palladium coating, observed by SEM (JEOL-6700F, Tokyo, Japan).

### Histological evaluations

As above specified, after 3-D culture, all the specimens were immersed in a fixative solution of 10% buffered formalin at pH 7.2 with 0.1 M sodium phosphate, for 4 h at room temperature and left overnight at 48°C. The specimens were processed to obtain thin ground sections with the Precise 1 Automated System (Assing, Rome, Italy). The specimens were dehydrated in an ascending series of alcohol rinses and embedded in a glycolmethacrylate resin (Techonovit 7200 VLC; Kulzer, Wehrheim, Germany). After polymerization, the specimens were sectioned along their longitudinal axis with a high-precision diamond disc to about 150 µm and ground down to about 30 µm with a specially designed grinding machine. The slides were stained with alizarin red or toluidine blue. The slides were observed in normal transmitted light under a Leitz-Laborlux microscope (Laborlux S, Leitz, Wetzlar, Germany). The histomorphometry was performed using a light microscope (Laborlux S, Leitz) connected to a high-resolution video camera (3CCD JVC KYF55B), and interfaced to a monitor and personal computer. This optical system was associated with a digitizing pad (Matrix Vision GmbH) and a histometry software package with image capturing capabilities (Image- Pro Plus 4.5; Media Cybernetics Inc., Milan, Italy). Experiments were performed in quadruplicate.

### Alizarin Red staining

For Alizarin Red staining, fixed cells or histological sections were incubated for 10 min at room temperature in a solution containing 1% Alizarin Red pH 4.3. Specimens were then washed with distilled water and viewed under the light microscope.

### Semi-quantitative RT-PCR analysis

Total RNA was extracted from specimens at 7, 15 and 30 days using TRI Reagent (Sigma, Milan, Italy), following the manufacturer's instructions, treated with DNase (Promega) to exclude DNA contamination and stored at −80°C until the assays. cDNA synthesis was carried out from total RNA (1 µg) using Superscript II reverse transcriptase (Invitrogen Celbio Italy, San Giuliano Milanese, Milan, Italy), and random primers. PCR analyses were carried out in triplicate using a TC-312 thermal cycler (Techne, Burlington, NJ, USA), in which samples underwent a 2-minute denaturing step at 94°C, followed by 35 cycles of 94°C for 30 s, 54–60°C for 60 s, 72°C for 30 s, and a final extension step at 72°C for 4 minutes. Each PCR reaction was performed in a total volume of 25 µl containing Tris buffer 10 mM pH 8, 0.2 mM of each dNTP, 1.5 mM MgCl2, and 0.2 µM of each primer, Taq DNA polymerase 1 U and 1 µl of each cDNA. The primer sequences were as follows: RUNX-2 forward, 5′-CACTCACTACCACACCTACC-3′, reverse 5′-TTCCATCAGCGTCAACACC-3′; Osterix forward-5′-GCAAAGCAGGCACAAAGAAG-3′, reverse 5′-AGGGAATGAGTGGGAAAAGG-3′; Osteocalcin forward 5′-CATGAGAGCCCTCACA-3′, reverse 5′-AGAGCGACACCCTAGAC-3′; VEGF forward 5′-TGACAGGGAAGAGGAGGAGA-3′, reverse 5′CGTCTGACCTGGGGTAGAGA-3′; BMP-2 forward 5′-CGTGTCCCCGCGTGCTTCTT-3′, reverse 5′-GGCTGACCTGAGTGCCTGCG-3′; Osteonectin forward 5′-AAACCCCTCCACATTCCC-3′, reverse 5′-ATTTTCCGCCACCACCTC-3′; Osteopontin forward 5′-GCCGAGGTGATAGTGTGGTT-3′, reverse 5′–TGAGGTGATGTCCTCGTCTG-3′; BAP forward 5′-TCAAACCGAGATACAAG CAC -3′, reverse 5′-GGCCAGACCAAAGATAGAGT -3′; BSP forward 5′- GGGCAGTAGTG ACTCATCCG -3′, reverse 5′- TTCTCAGCCTCAGAGTCTTCA- 3′. GAPDH was used as an internal control. The amplification products were separated on a 2% agarose gel in Tris-acetate EDTA (TAE) buffer. The RT-PCR experiments were made in triplicate.

### ELISA assay

In order to evaluate BMP-2 and VEGF levels in the culture medium, the complete supernatant medium was collected from cultures after 7, 15, 30 days from DPSCs and osteoblasts (*n = *9), cultured on different surfaces (Biocoral or flask surface). After centrifugation to remove particulates, 2 ml aliquots were stored at −20°C. After thawing at room temperature, 0.5 ml were collected from the aliquots and analyzed with an ELISA kit for BMP-2 or anti-VEGF (R&D, Milan, Italy). The ELISA experiments were made in triplicate.

### Statistical analysis

ANOVA test was used for statistical evaluation. Level of significance was set at p<0.05.
